# Inhibition of cathepsin K promotes osseointegration of titanium implants in ovariectomised rats

**DOI:** 10.1038/srep44682

**Published:** 2017-03-17

**Authors:** Chun Yi, Ke-Yi Hao, Ting Ma, Ye Lin, Xi-Yuan Ge, Yu Zhang

**Affiliations:** 1 Department of Oral Implantology, Peking University School and Hospital of Stomatology, Beijing 100081, People’s Republic of China; 2 Central Laboratory, Peking University School and Hospital of Stomatology, Beijing 100081, People’s Republic of China

## Abstract

The bone mineral deficiency in osteoporosis poses a threat to the long-term outcomes of endosseous implants. The inhibitors of cathepsin K (CatK) significantly affect bone turnover, bone mineral density (BMD) and bone strength in the patients with osteoporosis. Therefore, we hypothesised that the application of a CatK inhibitor (CatKI) could increase the osseointegration of endosseous implants under osteoporotic conditions. Odanacatib (ODN), a highly selective CatKI, was chosen as the experimental drug. Sixteen rats were randomised into 4 groups: sham, ovariectomy (OVX) with vehicle, OVX with low-dose ODN (5 mg/kg) and OVX with high-dose ODN (30 mg/kg). Titanium implants were placed into the distal metaphysis of bilateral femurs of each OVX rat. After 8 weeks of gavaging, CatKI treatment increased the removal torque, BMD and bone-to-implant contact (BIC). Moreover, high-dose CatKI exerted a better influence than low-dose CatKI. Furthermore, CatKI treatment not only robustly suppressed CatK gene (*CTSK*) expression, but also moderately reduced expression of the osteoblast-related genes *Runx2, Collagen-1, BSP, Osterix, OPN, SPP1* and *ALP*. Thus, CatKI could affect the osteoblast-related genes, although the balance of bone turnover was achieved mainly by CatK inhibition. In conclusion, CatKI prevented bone loss and aided endosseous implantation in osteoporotic conditions.

Osteoporosis, characterised by low bone mass and bone deterioration, increases bone fragility and the risk of fracture^1^,^2^. Sex hormones are crucial for maintaining bone mass, and the lack of oestrogen decreases bone mass and increases the risk of osteoporosis. Currently, approximately 40–70% of post-menopausal women develop osteoporosis in the United States, and more than 200 million people are affected by osteoporosis worldwide^3^.

Osseointegration refers to the direct contact between bone and implant, without any intervening soft tissue^4^. Osseointegrated implants are widely used for direct anchorage of dental prostheses in the jaw^5^ and hearing aids in the temporal bone^6^, and are an attractive bone-anchored treatment alternative for amputees^7^. During the osseointegration process, bone regeneration occurs with accumulation of osteoblast precursors, formation of bone matrix and mineralisation^8^. Therefore, deficiency of bone mass and destruction of bone matrix, caused by osteoporosis, hamper the osseointegration. Previous studies reported that osseointegration was inhibited by osteoporosis, which in turn led to increased mobility and eventual implant loss. Therefore, osteoporosis has been considered a relative contraindication for endosseous implant surgery^9^,^10^,^11^,^12^,^13^,^14^,^15^, and endosseous implantation in patients with osteoporosis is still problematic.

Owing to the severe prevalence of osteoporosis, a few standard antiresorptive therapies for osteoporosis, such as bisphosphonates, calcitonin, selective oestrogen receptor modulators and oestrogen emerged gradually^16^. However, even the most popular therapies have shown subsequent adverse effects. For example, bisphosphonates have been linked to painful refractory osteonecrosis in the bone^17^,^18^. Thus, the safety of implant restoration in patients treated with bisphosphonates is still debated^19^.

Bone comprises approximately 60% calcium hydroxyapatite and 40% protein. The majority of the protein is collagen I type. In order to degrade the bone effectively, osteoclasts secrete acid to demineralise hydroxyapatite and a lysosomal cysteine protease—cathepsin K (CatK)—to degrade the bone matrix proteins^16^,^20^. Overexpression of CatK gene (*CTSK*) can increase the bone turnover rate, leading to low bone mass^20^,^21^,^22^. In addition, odanacatib (ODN), a highly selective and sensitive inhibitor of CatK, exhibits high activity and selectivity, indicating its efficacy for treatment of osteoporosis^23^.

A previous study implicated the long-term decrease in bone turnover and antiangiogenic activity due to bisphosphonates in the development of osteonecrosis^24^. However, unlike bisphosphonates, cathepsin K inhibitor (CatKI) reduces the resorption efficiency of cells, but does not change the other normal osteoclastic functions such as differentiation, migration and polarisation^23^,^25^. A previous study showed that the treatment-related responses of bone resorption markers were similar for CatKI and the typical bisphosphonate alendronate (ALN); however, the reduction in the levels of bone-formation markers was less with CatKI than with ALN^26^. Moreover, CatKI was generally well tolerated without bone necrosis^27^. Thus, CatK is considered an important target for the treatment of postmenopausal osteoporosis^28^.

Considering the possible benefits of CatKI, we believe that osseointegrated implants can be successfully improved with post-implantation CatKI treatment in patients with osteoporosis. To provide evidence for this hypothesis, we aimed to assess the effect of CatKI on bone formation and osseointegration in rats that underwent ovariectomy (OVX) and explore the molecular mechanisms of CatKI. As a highly selective inhibitor of CatK, ODN was chosen as the experimental drug.

## Results

### Confirmation of osteoporotic bone condition

**Figure 1:**
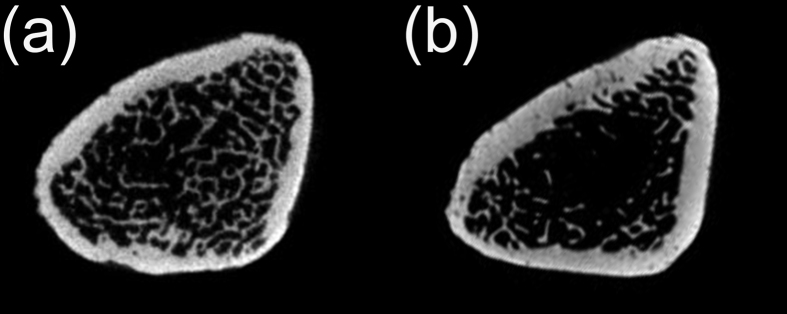
Confirmation of osteoporotic bone condition. The images compare the sham-operated rats (**a**) and OVX rats (**b**); the latter has less trabecular bone, expanded marrow cavities and disorganised trabecular architecture. OVX, ovariectomy.

Micro-computed tomography (micro-CT) showed that OVX rats had less trabecular bone, disorganized trabecular architecture and expanded marrow cavities compared to the sham-operated animals (Fig. 1).

### Removal torque testing

**Figure 2:**
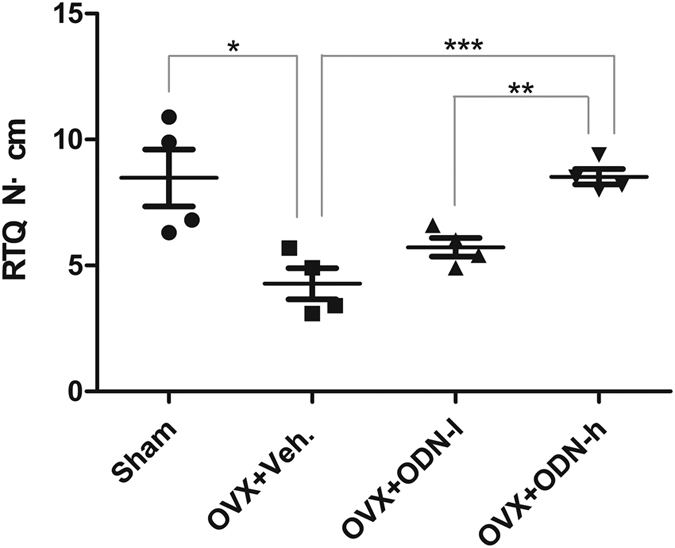
Strength of bone-implant integration expressed by the removal torque values for over 8 weeks. Significant differences are observed between the OVX + ODN-h and OVX + Veh groups, and the values in the OVX + ODN-h group are significantly higher than those in the OVX + ODN-l group (*p < 0.05; **p < 0.01; ***p < 0.001). ODN, odanacatib; OVX, ovariectomy; ODN-h, high-dose ODN (30 mg/kg); ODN-l, low-dose ODN (5 mg/kg); Veh, vehicle.

Data from the removal torque test of groups that received different treatments after surgery are presented in Fig. 2. The *t-*test showed significant differences between the high-dose ODN (OVX + ODN-h) group and the vehicle (OVX + Veh) group (*p* < 0.001). Moreover, significant differences were observed between the OVX + ODN-h group and the low-dose ODN (OVX + ODN-l) group. Although removal torque values in the OVX + ODN-l group were higher than those in the OVX + Veh group, there were no statistically significant differences between them.

### Micro-CT evaluation

**Figure 3:**
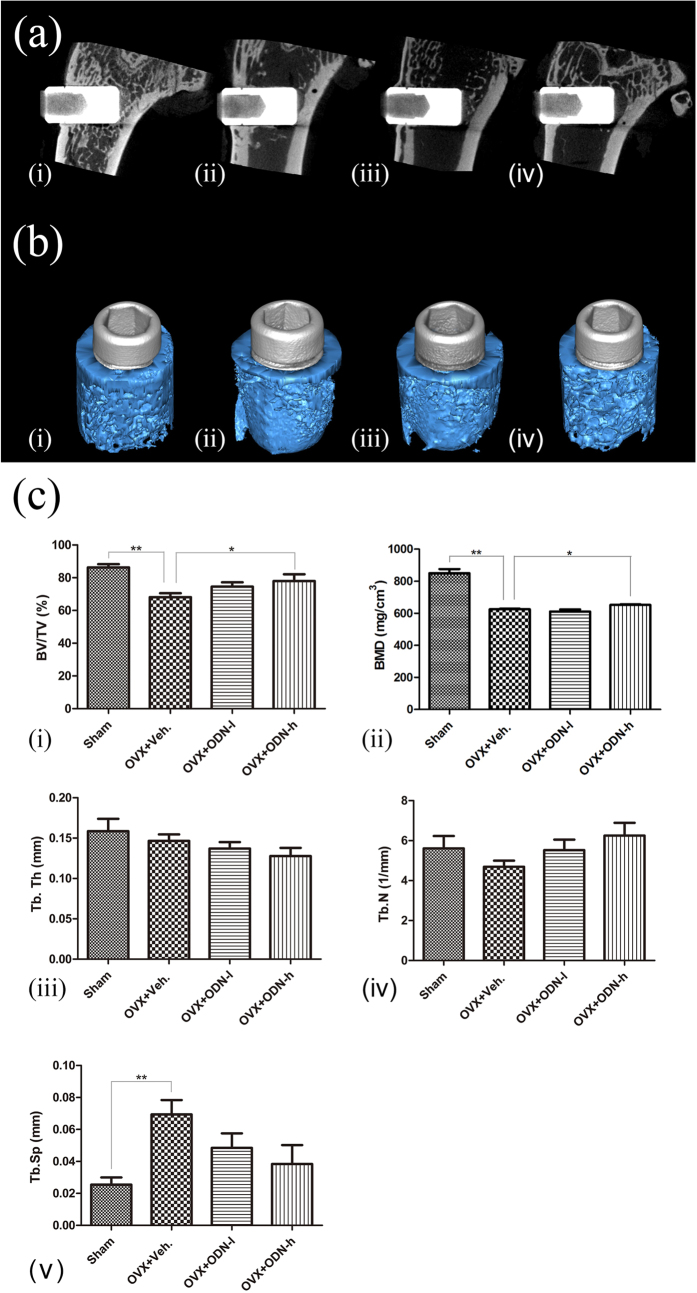
Improvements in bone quantity and bone mineral density shown by micro-computed tomography and the corresponding 3D models. (**a**) Representative sections of micro-computed tomography images and (**b**) the corresponding 3D models show the differences in bone mass between the 4 groups: sham group (i), OVX + Veh group (ii), OVX + ODN-l group (iii) and OVX + ODN-h group (iv); the grey cylinders represent implants and blue portions represent bone. (**c**) After CatKI application, the BV/TV (i) and BMD (ii) of the OVX + ODN-h group are significantly higher than those of the OVX + Veh group. The Tb.Th (iii) and Tb.Sp (v) show a decrease with the increased dose, but the Tb.N (iv) shows an increase (*p < 0.05; **p < 0.01). ODN, odanacatib; OVX, ovariectomy; ODN-h, high-dose ODN (30 mg/kg); ODN-l, low-dose ODN (5 mg/kg); Veh, vehicle; BMD, bone mineral density; Tb.Th, trabecular thickness; Tb.N, trabecular number; Tb.Sp, trabecular separation.

The 4 representative images of micro-CT and the corresponding 3D models are shown in Fig. 3; the images indicate the differences in bone formation around the implants. The bone volume/total volume (BV/TV) and bone mineral density (BMD) of the OVX + ODN-h group was significantly higher than that of the OVX + Veh group (*p* < 0.05). However, treatment with the low-dose CatKI led to a numerical, but not statistically significant, increase in the BV/TV. Further, no significant difference in the BV/TV and BMD was observed between the OVX + ODN-l and OVX + ODN-h groups. To obtain sufficient data for analysis, trabecular thickness (Tb.Th), trabecular number (Tb.N) and trabecular separation (Tb.Sp) were measured together. After CatKI administration, the Tb.Th and Tb.Sp decreased, but the Tb.N increased with high-dose CatKI.

### Histomorphometric analysis

**Figure 4:**
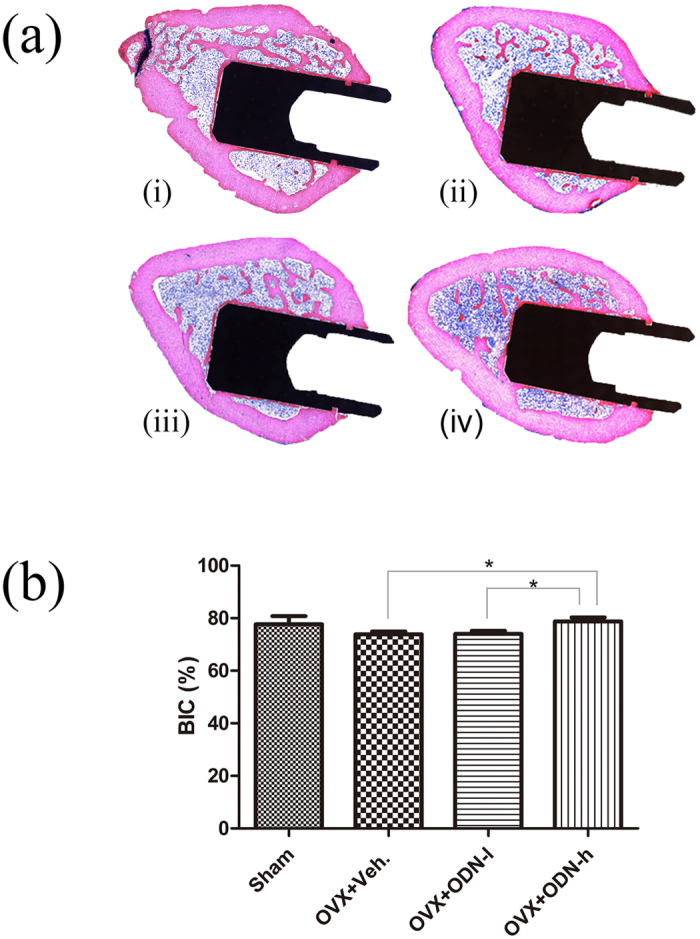
Light microscopy images and the BIC percentages determined by histomorphometric analysis. (**a**) Light microscopy images of the (i) sham group, (ii) OVX + Veh group, (iii) OVX + ODN-l group, and (iv) OVX + ODN-h group show differences in new-bone formation and osseointegration. (**b**) Comparison of the BIC percentages determined by histomorphometric analysis shows significant differences between the OVX + Veh group and OVX + ODN-h group and between the OVX + ODN-l group and OVX + ODN-h group (*p < 0.05). ODN, odanacatib; OVX, ovariectomy; ODN-h, high-dose ODN (30 mg/kg); ODN-l, low-dose ODN (5 mg/kg); Veh, vehicle; BIC, bone-to-implant contact.

A numerically significant difference was observed between the OVX + ODN-h and OVX-Veh groups (*p* < 0.05; Fig. 4). A significant difference was also noted between the two CatKI-treated groups (*p* < 0.05). These results demonstrated that bone-to-implant contact (BIC) decreased after OVX surgery but significantly increased when CatKI was administered. However, the BIC of the low-dose group was almost the same as that of the OVX-Veh group. Moreover, new bone with more direct contact was formed around the implants in the OVX + ODN-h group, which was nearly similar to that seen in the Sham group. These findings were confirmed by digital light microscopy images (Fig. 4).

### Real-time polymerase chain reaction (RT-PCR) analysis

**Figure 5:**
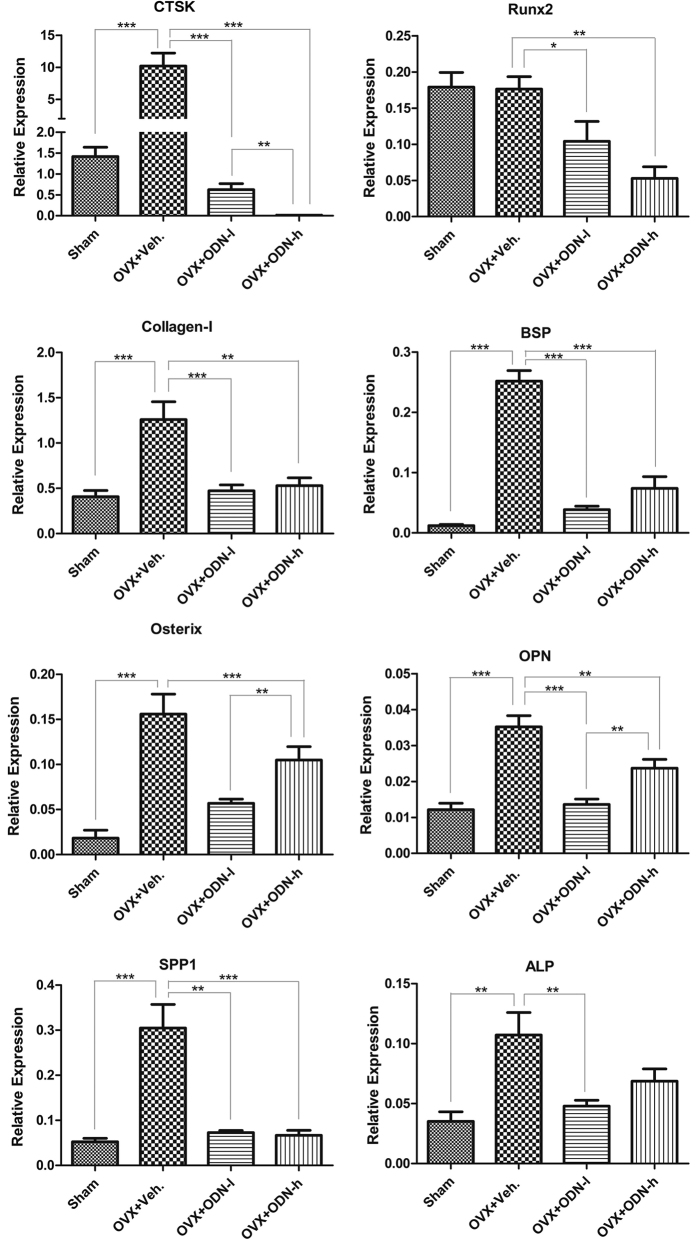
Effect of CatKI on the expressions of *CTSK, Runx2, Collagen-1, BSP, Osterix, OPN, SPP1* and *ALP* in the bone tissue around implants. Data from the expression analysis of the selected key genes known to be related with osteoblast are shown (*p < 0.05; **p < 0.01; ***p < 0.001). CatKI, cathepsin K inhibitor.

Gene expressions of several bone-specific markers were analysed by RT-PCR. After ODN application, the expression of *CTSK* diminished remarkably (*p* < 0.001; Fig. 5). The expressions of *Runx2, Collagen-1, BSP, Osterix, OPN* and *SPP1* were significantly lower in the OVX + ODN-h group than in the OVX + Veh group (*p* < 0.01). Compared with the OVX + Veh group, the expressions of *Collagen-I, BSP, Osterix, OPN* and *ALP* reduced in the OVX + ODN-l group, but were upregulated in the OVX + ODN-h group. In addition, the expressions of these bone-formation markers increased after OVX treatment, but decreased in the CatKI groups.

## Discussion

In the past, the number of cases of bisphosphonate-related osteonecrosis increased gradually to an alarming number; however, CatKI is generally well tolerated without bone necrosis. The efficacy of CatKI on the multiple bone sites in monkeys has been previously reported^20^. To assess the role of CatKI in osseointegration, we measured the BIC, BMD and bone strength around the endosseous implants of titanium in OVX rats.

Our study showed that CatKI treatment significantly prevented bone loss, enhanced bone formation and implant osseointegration and balanced the expression of bone-related genes. In addition, CatKI administration decreased the Tb.Th and Tb.Sp, and increased the BV/TV, BMD and Tb.N. Furthermore, it improved the BIC, thereby confirming the positive influence of CatKI on osseointegration. Moreover, the removal torque values showed an evident increase after CatKI treatment. The high-dose group exhibited greater potential to promote osseointegration than the low-dose group. Moreover, the effect of CatKI on the removal torque values and BIC observed in this study is comparable to that of ALN noted in the previous studies^29^,^30^.

In our study, CatKI treatment increased the bone mass and BMD, and improved the BIC, resulting in high removal torque values and bone strength^31^,^32^. A previous study found a significant linear correlation between immediate BIC and BMD at the placement site^33^, which explains the increase in the initial BIC caused by the high BMD in this study. In addition, the average torque values, which reflect bone strength, are known to be significantly correlated with histological bone density data^34^.

Several bone formation-related factors were selected and analysed in the present study. With regard to gene expression of both bone formation-related and bone resorption-related factors, there were significant differences between the sham group and the OVX + Veh group. After CatKI treatment, most values in the CatKI groups were close to the levels of the sham group, which indicated that CatKI administration could restore the values to the normal level.

The OVX + Veh group showed a higher expression of osteoblastic genes than the sham group, indicating an increased bone turnover. After OVX, bone resorption is activated by oestrogen deficiency, and bone formation increases to fill the resorption cavities^35^. In the present study, compared with the OVX + Veh group, the osteoblastic genes in all groups were downregulated after CatKI treatment. Interestingly, compared with the low-dose group, most bone formation-related gene expressions were subsequently upregulated in the high-dose group. In line with this finding, a previous study that compared ODN and ALN showed that the patterns of treatment-related decrease in bone-formation markers had an inverse dose-dependent relationship with ODN treatment^26^.

**Figure 6:**
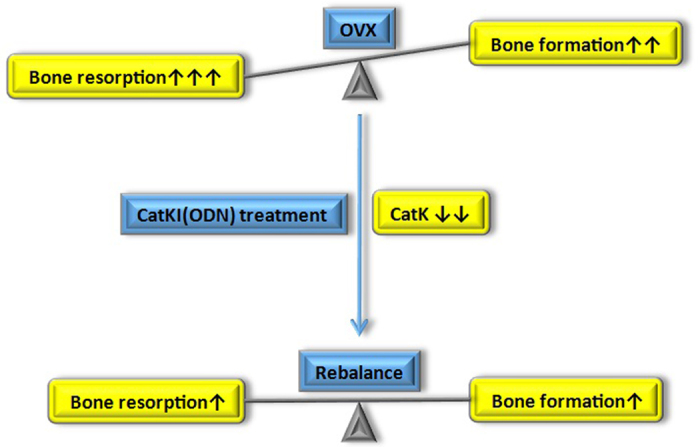
Schematic illustration of the role of CatKI in osseointegration of titanium implants in ovariectomised rats. After OVX, the bone resorption is activated by oestrogen deficiency, and bone formation increases to fill the resorption cavities. CatKI robustly suppresses bone resorption and decreases the expression of osteoclastic genes by inhibiting CatK. To rebalance bone turnover, the level of bone formation and the compensatory overexpression of osteoblastic genes decreases moderately. However, the additive effects where bone formation exceeds bone resorption promote the bone quality and quantity, which leads to high BMD and increased osseointegration. CatK, cathepsin K; CatKI, cathepsin K inhibitor; BMD, bone mineral density; OVX, ovariectomy.

Osteoclastic bone resorption and osteoblast-mediated bone formation are coupled in the mature skeleton^36^. Following OVX surgery in this study, osteoporotic rodent models were successfully established, and the expressions of osteoclastic genes increased. To rebalance bone regeneration and bone resorption, the expressions of osteoblastic genes were also upregulated. However, CatKI inhibited the bone turnover, blocked bone loss and decreased the expression of osteoclastic genes. Alongside, the compensatory overexpression of osteoblastic genes decreased. The expression of the osteoblastic genes of the OVX + ODN-l group seemed to be downregulated and tended to be similar to that of the sham group. All the abovementioned variations demonstrate that CatKI has the potential to regulate and rebalance bone regeneration and bone resorption (Fig. 6).

Osseointegration is a long, dynamic process wherein bone remodelling plays an important role after implant surgery. The relative expression of *CTSK* in the vehicle group was 16.33 times that in the low-dose group and 885.74 times that that in the high-dose group. Regarding bone formation, the expressions of the osteoblastic genes were diminished after CatKI application, but the relative expressions of all osteoblastic genes in the vehicle group only were several times higher than that in the low-/high-dose groups. For example, the relative expression of *Runx2* in the vehicle group was 1.70 times higher than that in the low-dose group and 3.34 times higher than that in the high-dose group. Based on these findings, we concluded that CatKI not only moderately and transiently inhibits bone formation, but also downregulates expression of bone resorption-related genes more robustly^25^. Some other studies on CatKI application have also drawn the same conclusions^37^. Hence, these additive effects promote bone formation, which leads to a high BMD and osseointegration. The imbalance in bone turnover, where bone formation exceeds bone resorption, is the currently accepted mechanism for CatKI action. Moreover, in a phase 2 trial, bone resorption markers remained low for 5 years after CatKI application^38^. Although the levels of bone-formation markers initially decrease, they return to the near-baseline levels within 2 years of continued therapy^39^,^40^.

In our study, there was a significant difference between the vehicle and low-dose groups in the gene-expression pattern, but no significant difference was noted at the tissue and functional levels. Changes in the gene-expression pattern were initial factors that resulted in subsequent changes in tissue and functional levels. Since the CatKI administration period was only 8 weeks, the time interval may not have been sufficient for the low-dose group to show changes in the histological and functional levels. Compared to the low-dose group, the high-dose group showed stronger inhibition of *CTSK*. Therefore, we found a significant difference in the gene-expression pattern at the tissue and functional levels in the vehicle and high-dose group.

Thus far, the effects of CatKI on the endosseous implants in OVX rats have not been reported. Although our results provided evidence for the efficacy of CatKI administration, there were some limitations to our findings. Firstly, we did not obtain data for the protein levels by western blot. Secondly, we were unable to confirm the capacity of CatKI by *in vitro* experimentation to explain the mechanism in depth. Thirdly, the increased risk of stroke, atrial fibrillation and other side effects hampered the development of ODN, which was the experimental drug used in this study. In Sept 2016, Merck officially decided to discontinue ODN development due to its bio-safety concerns^41^. However, another study determined and compared the structures of inhibitor-free mouse CatK, human CatK and ODN bound to human CatK. In Jan 2017, the researchers revealed that their findings could be used to design a transgenic mouse, which would be highly beneficial to study the observed adverse effects of CatKI in human trials and explore ways to avoid these effects^42^. Therefore, CatKI can be used as a potential target for endosseous implantation in osteoporotic conditions. However, there is a long way to go for its clinical applications.

In conclusion, our study showed that the application of CatKI could increase osseointegration, prevent bone loss and benefit endosseous implantation in osteoporotic conditions.

## Methods

### Animal care and grouping

**Figure 7:**
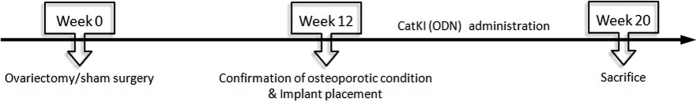
Study design. The time frame of the experimental workflow shows the sequence of OVX surgery, confirmation of the OVX model, implant insertion, sacrifice and duration of CatKI administration. CatKI, cathepsin K inhibitor; OVX, ovariectomy.

Sixteen, 8-month-old, female Sprague–Dawley (SD) rats (weight, 385 ± 55 g) were given water and soft diet food *ad libitum* in a temperature-controlled environment with regular 12-h cycles of light and dark. The rats were randomised into 4 groups, with 4 rats in each group: sham group, OVX + Veh group, OVX + ODN-l group and OVX + ODN-h group. This study was approved by the Animals Ethics Committee of the Peking University Health Center (LA2012-11). The experiments were performed in accordance with the approved guidelines and regulations. A chart comprising the experimental design is presented in Fig. 7.

### OVX procedure

Surgeries were performed under intraperitoneal anaesthesia with 40 mg/kg sodium pentobarbital (Sigma-Aldrich, St. Louis, MO, USA). In the case of the sham-operated animals, the adipose tissue around ovaries was exteriorised gently. In the OVX groups, the rats received bilateral OVX via a 2-cm back incision^43^.

### Assessment of osteoporotic condition

Twelve weeks after the OVX procedure, 3 animals of the sham group and 3 animals of the OVX experimental groups were anesthetised and scanned by micro-CT (Siemens, Munich, Germany).

### Implant surgery

**Figure 8:**
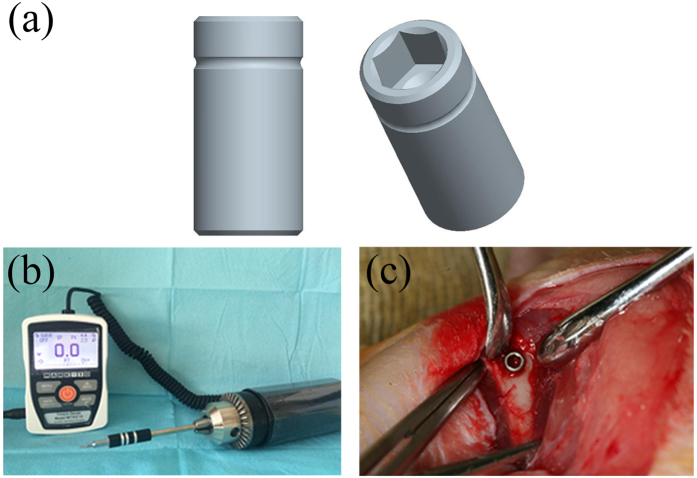
The pattern of the experimental implants, the engaging torque-testing machine and the intraoperative location of implants. (**a**) The pure titanium cylindrical implants. (**b**) The torque-testing machine. (**c**) The implants are placed into the distal metaphysis of bilateral femurs where the notch is at the bone level.

Implant surgery was performed after successful establishment of the OVX model. The female SD rats were anesthetised as mentioned above in ‘OVX procedure’, and pure titanium cylindrical implants (Wego, Weihai, China) with smooth surfaces (2.0 mm in diameter and 4.0 mm in length) were placed into the distal metaphysis of bilateral femurs following the standardised protocol (Fig. 8), such that each animal received two implants in the legs. To prevent the influence of self-tapping and bone condensing of the thread surface, these implant surfaces were designed to be smooth. The notches on them were used to ensure the consistency in the implanted depth, and the inner hexagonal structure was designed to fit the engaging torque-testing machine (Model MTT03–12 digital torque gauge; Mark 10 Corporation, NY, USA).

### Postoperative treatment and sacrifice

Following implant insertion, ODN (5 mg/mL) was administered to the OVX + ODN-l and OVX + ODN-h groups at concentrations of 1 mL/kg and 6 mL/kg, respectively, by gavaging once a day for 8 weeks. The OVX + Veh group was gavaged with 0.5% sodium carboxymethyl cellulose (Sigma-Aldrich) at a concentration of 6 mL/kg over the same duration. After the gavage administration, the rats of each group were sacrificed by injecting sodium pentobarbital intravenously. The implants were harvested and fixed in 10% buffered formalin together with the surrounding bone.

### Removal torque testing

Immediately after the sacrifice, the left femur of each animal was subjected to removal torque testing to determine the necessary force to extract the implant from the bone. This biomechanical test was used to specifically measure the strength of bone-implant integration, which reflects the potential effect of CatKI on osseointegration. The engaging torque-testing machine was operated automatically. The peak torque values to initiate reverse rotation were measured and recorded. To reduce the deviation, all the tests were performed by the same operator.

### Micro-CT evaluation

Following the biomechanical testing, another unilateral femur of each animal was prepared for the micro-CT scanning. Micro-CT was performed using an Inveon MM system (Siemens, Munich, Germany). Specimens were located and scanned in whole, with 360° rotation in 360 equiangular steps. Images were acquired at an effective pixel size of 8.99 μm, voltage of 80 kV, current of 500 μA and exposure time of 1500 ms. The images consisted of 1024 slices, with a voxel size of 8.99 μm × 8.99 μm × 8.99 μm. Two-dimensional images were used to construct 3D reconstructions using Inveon Research Workplace 3.0 software (Siemens). After acquiring the 3D images, the peri-implant volume-of-interest of 1 mm was established manually, and the threshold value was adjusted appropriately to distinguish trabecular bone from bone marrow. Inveon Research Workplace 3.0 software (Siemens) was used to automatically compute BV/TV, BMD and the following trabecular bone morphological parameters: Tb.N, Tb.Sp and Tb.Th^44^,^45^.

### Histomorphometric analysis

The femoral specimens were progressively dehydrated in increasing concentrations of alcohol at increments of 10% from 70% to 100%. Subsequently, they were embedded in methylmethacrylate (Sigma-Aldrich). Only one slice in the longitudinal direction of each implant was prepared by the Exakt Cutting and Grinding equipment (Exact Apparatebau, Norderstedt, Germany). Further, the histological sections were stained with methylene blue-acid fuchsine staining method^46^. Following that, the sections were analysed using a digitised image-analysis system (Leica Imaging System, Cambridge, England). The peri-implant region-of-interest of 200 μm was established manually. The percentages of BIC were calculated using the BIOQUANT OSTEO Bone Biology Research System (BIOQUANT Image Analysis Corporation, TN, USA) as the ratio of the length of the direct-contact new bone to the outer perimeter of the intrabony implant^47^ (Fig. S1).

### RNA isolation and real-time PCR analysis

Peri-implant bone was obtained from the left femur and stored immediately in freezing tubes. All the samples were preserved and triturated in liquid nitrogen. Total RNA from the implant surface and the surrounding bone was isolated with TRIZOL Reagent (Invitrogen, Grand Island, NY, USA). A total of 2 μg total RNA was reverse-transcribed into cDNA using the Superscript First-Strand Synthesis System (Invitrogen) as per the manufacturer’ s protocol. Reactions were conducted in a 20-μL reaction mixture with the FastStart SYBR Green Master (Roche, Shanghai, China) using the ABI 7500 real-time PCR detection system (ABI, Thermo Fisher, Waltham, MA, USA). The expression of genes related to bone metabolism was normalised to β-actin expression, expressed as 2^−(ΔCt)^. Subsequently, PCR amplification (40 cycles of 95 °C for 15 s and 60 °C for 60 s) was conducted, and the melting curves were recorded and analysed. Primers for the genes of bone resorption and formation are listed in Table 1.

### Statistical analysis

Statistical analysis was performed using SPSS Statistics v19 (SPSS Inc., Chicago, IL, USA). The statistical data are presented as mean and standard deviations. The *t-*test was used to compare the vehicle group and the other three groups, and compare the influence of CatKI with different concentrations. A *p*-value < 0.05 was considered statistically significant.

## Additional Information

**How to cite this article:** Yi, C. *et al*. Inhibition of cathepsin K promotes osseointegration of titanium implants in ovariectomised rats. *Sci. Rep.* **7**, 44682; doi: 10.1038/srep44682 (2017).

**Publisher's note:** Springer Nature remains neutral with regard to jurisdictional claims in published maps and institutional affiliations.

## Supplementary Material

Supplementary Information

## Figures and Tables

**Table 1:** The forward and reverse primers of each gene analysed.

Gene	Forward primers (5′-3′)	Reverse primers (5′-3′)
*CTSK*	GTT ACT CCA GTC AAG AAC CAG G	TCT GCT GCA CGT ATT GGA AGG
*Runx2*	GAC TGT GGT TAC CGT CAT GGC	ACT TGG TTT TTC ATA ACA GCG GA
*Collagen-1*	TCT GAC TGG AAG AGC GGA GAG	GAG TGG GGA ACA CAC AGG TCT
*BSP*	CCG GCC ACG CTA CTT TCT T	TGG ACT GGA AAC CGT TTC AGA
*Osterix*	CAT CTA ACA GGA GGA TTT TGG TTT G	AAG CCT TTG CCC ACC TAC TTT T
*OPN*	CAC TCC AAT CGT CCC TAC AGT	CTG GAA ACT CCT AGA CTT TGA CC
*SPP1*	AGA GCG GTG AGT CTA AGG AGT	TGC CCT TTC CGT TGT TGT CC
*ALP*	CCT AGA CAC AAG CAC TAA CAC TA	GTC AGT CAG GTT GTT CCG ATT C
