# Supplementary material for: Inhibition of cathepsin K promotes osseointegration of titanium implants in ovariectomised rats
Source: Sci Rep. 2017 Mar 17;7:44682. doi: 10.1038/srep44682 (PMC5356343; doi:10.1038/srep44682)
Supplement: Supplementary Information [file srep44682-s1.doc]

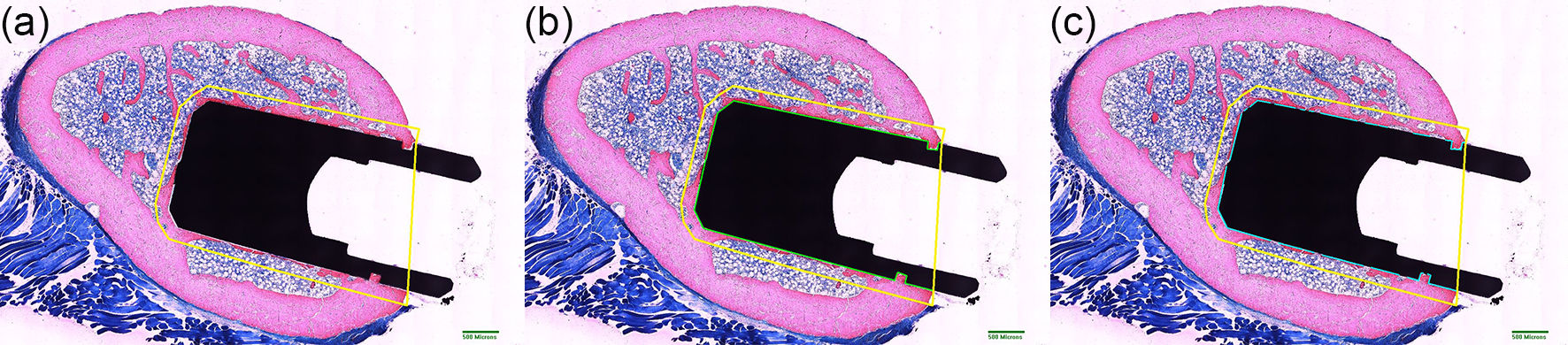


**Supplementary Figure S1: The calculation procedure for BIC**

BIC was calculated and recorded as a proportion of the length of the bone surface border that was in direct contact with the outer perimeter of the intrabony implant. Firstly, the peri-implant ROI of 200 μm was established manually (a) (yellow line represents ROI). Scale bar = 500 μm. Secondly, the outer perimeter of the intrabony implant was marked and calculated (b) (green line represents outer perimeter). Thirdly, we marked the inner margin of direct-contact bone and calculated the length (c) (blue line represents inner margin). Finally, BIC was calculated as the ratio of the length of the direct-contact new bone to the outer perimeter of the intrabony implant.

BIC, bone to implant contact; ROI, region-of-interest

**Manuscript Title:** Inhibition of cathepsin K promotes osseointegration of titanium implants in ovariectomised rats

**Author List:** Chun Yi, Ke-Yi Hao, Ting Ma, Ye Lin, Xi-Yuan Ge*, Yu Zhang*

*Address correspondence to [zhang76yu@163.com](mailto:zhang76yu@163.com) (Yu Zhang); [gexiyuan@ bjmu.edu.cn (Xi-Yuan](mailto:gexiyuan@sina.com(Xi-Yuan) Ge)
